# Discovery of Novel ROCK1 Inhibitors *via* Integrated Virtual Screening Strategy and Bioassays

**DOI:** 10.1038/srep16749

**Published:** 2015-11-16

**Authors:** Mingyun Shen, Sheng Tian, Peichen Pan, Huiyong Sun, Dan Li, Youyong Li, Hefeng Zhou, Chuwen Li, Simon Ming-Yuen Lee, Tingjun Hou

**Affiliations:** 1 College of Pharmaceutical Sciences, Zhejiang University, Hangzhou, Zhejiang 310058, China; 2 State Key Lab of CAD&CG, Zhejiang University, Hangzhou, Zhejiang 310058, China; 3 Institute of Functional Nano & Soft Materials (FUNSOM), Soochow University, Suzhou, Jiangsu 215123, China; 4 State Key Laboratory for Quality Research in Chinese Medicine and Institute of Chinese Medical Sciences, University of Macau, MO 999078, China

## Abstract

Rho-associated kinases (ROCKs) have been regarded as promising drug targets for the treatment of cardiovascular diseases, nervous system diseases and cancers. In this study, a novel integrated virtual screening protocol by combining molecular docking and pharmacophore mapping based on multiple ROCK1 crystal structures was utilized to screen the ChemBridge database for discovering potential inhibitors of ROCK1. Among the 38 tested compounds, seven of them exhibited significant inhibitory activities of ROCK1 (IC_50_ < 10 μM) and the most potent one (compound TS-f22) with the novel scaffold of *4-Phenyl-1H-pyrrolo [2,3-b] pyridine* had an IC_50_ of 480 nM. Then, the structure-activity relationships of 41 analogues of TS-f22 were examined. Two potent inhibitors were proven effective in inhibiting the phosphorylation of the downstream target in the ROCK signaling pathway *in vitro* and protecting atorvastatin-induced cerebral hemorrhage *in vivo*. The high hit rate (28.95%) suggested that the integrated virtual screening strategy was quite reliable and could be used as a powerful tool for identifying promising active compounds for targets of interest.

Rho-associated kinases (ROCK1 and ROCK2) belong to the AGC family of serine-threonine kinases^1^,^2^,^3^,^4^,^5^, and catalyze the phosphorylation of several downstream targets, including myosin light chain (MLC), Lin-11 Isl-1 Mec-3 kinase (LIMK), ezrin/radixin/moesin (ERM), adducin, calponin, and collapsin response mediator protein-2 (CRMP-2), etc^6^,^7^. ROCK plays a crucial role in numerous cellular functions including cell contraction, actin organization, cell migration and proliferation^8^,^9^. Consequently, ROCKs have been regarded as promising targets for the treatment of cardiovascular diseases, neurological diseases and cancers^10^,^11^,^12^,^13^,^14^,^15^,^16^,^17^.

A certain number of ROCK inhibitors with different scaffolds have been discovered^18^,^19^,^20^,^21^, and they can be roughly classified into several categories: isoquinoline derivatives, indazole derivatives, urea derivatives, amino-pyrimidine derivatives, etc^22^. Until now, numerous ROCK inhibitors have been pushed into the clinical trials, including fasudil, Y39983, SAR407899, K115, AR12286, DE104, BA210, AMA0076, INS117548, etc^23^,^24^,^25^,^26^,^27^,^28^. But unfortunately, only fasudil has been used clinically in Japan since 1995 for the treatment of cerebral vasospasm and ischemia, and therefore, it is still urgent to discover novel inhibitors of ROCK1.

Experimental high-throughput screening (HTS) can evaluate huge chemical libraries against relevant biological targets in a relatively short time, but it is time-consuming and costly. Besides, not every assay can be applied automatically for HTS. As a powerful and complementary approach to experimental HTS, virtual screening (VS) has gained more and more attentions^29^,^30^. In most traditional applications of structure-based VS, a single rigid crystal structure of protein-ligand complex was usually used for molecular docking or structure-based pharmacophore search. However, the importance of protein flexibility to protein-ligand interaction has been widely recognized, and the ignorance of protein flexibility might have detrimental effect on the performance of VS^31^. Due to huge computational cost, it is not realistic to screen massive chemical libraries against all possible conformations of a target^32^, and the use of multiple receptor conformations (MRC) of a target for structure-based VS represents a balanced strategy in terms of costs and benefits^31^,^33^,^34^,^35^,^36^,^37^,^38^,^39^,^40^,^41^. For example, our previous studies showed that an integrated VS approach by combining molecular docking and pharmacophore mapping based on multiple representative conformations of a target yielded better performance than the docking-based VS based on any single rigid conformation^41^,^42^.

Recently, we reported the discovery of 12 potent ROCK1 inhibitors (IC_50_ values varying from 7 to 28 μM) by using *Glide* docking based on a single crystal structure of ROCK1^43^. However, in this study, the hit rate (inhibitor enrichment), defined as the proportion of the inhibitors found in the tested compounds (174), was only 6.90%. Apparently, the prediction accuracy of the docking-based VS based on a single structure of ROCK1 was not satisfactory. In this study, an integrated VS strategy based on multiple ROCK1 structures was utilized to screen the ChemBridge database, and 38 compounds were purchased and submitted to bioassays. The experimental results showed that 7 compounds exhibited strong inhibitory activities of ROCK1 with IC_50_ < 10 μM, and the most potent one (TS-f22) with the scaffold of *4-Phenyl-1H-pyrrolo [2,3-b] pyridine* had an IC_50_ of 480 nM. Then, the structure-activity relationships (SARs) for 41 analogues of TS-f22 were discussed. Finally, two compounds were tested in zebrafish, and they showed considerable protective effects against atorvastatin-induced cerebral hemorrhage.

## Methods

### Integrated Virtual Screening Protocol

**Figure 1:**
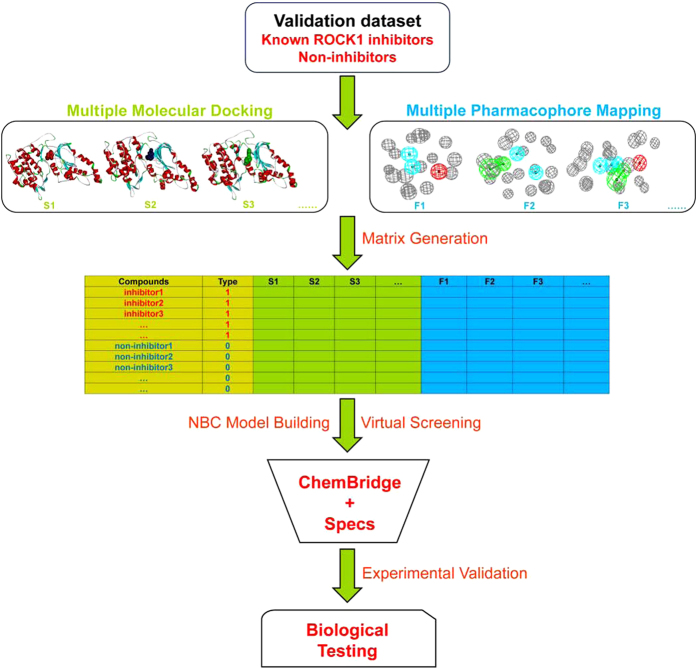
The workflow of the integrated VS protocol by combining molecular docking and pharmacophore mapping based on multiple crystal structures of ROCK1.

The integrated VS protocol based on multiple structures of ROCK1 was illustrated in Fig. 1, which was described in detail in our previous study^42^. First, nine crystal structures of ROCK1-ligand complexes were downloaded from RCSB protein data bank (PDB), and eight of them (PDB entries: 2ESM^44^, 3D9V^44^, 2V55^45^, 3NCZ^46^, 3NDM^46^, 3V8S^47^, 3TV7^48^, and 3TWJ^48^) that could satisfy the requirements of “docking power” (RMSD ≤ 2.0 Å) and “distinguishing power” (*P*-value ≤ 10^−20^) by using either *Glide* SP or XP scoring were used in VS^42^. For each complex, the *Protein Preparation wizard* in Schrödinger 9.0^49^ was used to remove all crystallographic water molecules, add hydrogen atoms, assign partial charges and minimize the structure until the root-mean-square deviation (RMSD) reached a maximum value of 0.3 Å. A dataset with the known inhibitors and non-inhibitors of ROCK1 was prepared to develop the integrated VS model. The 350 non-duplicated known inhibitors of ROCK1 were retrieved from the BindingDB database^50^. In order to mimic the unbalanced nature between known inhibitors and non-inhibitors, the ratio of non-inhibitors *versus* known inhibitors was set to 20. Consequently, 7000 presumed non-inhibitors were chosen from the ChemBridge database by using the *Find Diverse Molecules* protocol in Discovery Studio 3.1 (DS3.1)^51^. Then, each compound in the dataset was docked into the binding site of each crystal structure of ROCK1 by using *Glide* in Schrodinger 9.0 and scored by the Standard Precision (SP) or Extra Precision (XP) scoring mode^49^. The “docking power”, which measures the consistency between the predicted binding pose and the experimental structure of a ligand in the active site, and the “discrimination power”, which evaluate the capability of docking scores to distinguish the known inhibitors from non-inhibitors of ROCK1, were examined for each crystal structure of ROCK1-ligand complex.

Then, the complex-based pharmacophore models for eight ROCK1 complexes were generated by using the *Receptor-Ligand Pharmacophore Generation* protocol in DS3.1^51^. For each pharmacophore model, the minimum number of the pharmacophore features (interaction patterns) was set to 3, and the maximum number of the pharmacophore features was set to the same number of the total features that could match the protein-ligand interactions. For the crystal structure of each ROCK1-ligand complex, up to 10 pharmacophore models were generated and ranked by the selectivity. Besides, by using the *Conformation Generation* protocol in DS3.1^51^, the low-energy conformations of each molecule in the dataset were generated and the maximum number of the conformations *per* molecule was set to 100. The discrimination power of the pharmacophore models for each complex was also evaluated.

Consequently, each molecule in the dataset could be quantitatively assessed by eight docking scores calculated by *Glide* docking and eight fit values given by pharmacophore mapping^42^. Those docking scores and fit values were used as the independent variables (*X*), and 1/0 (1 for inhibitor and 0 for non-inhibitor) was used as the response variable (*Y*). Then, the naïve Bayesian classification (NBC) technique implemented in the *Create Bayesian Model* protocol in DS3.1^51^ was employed to develop classifiers for VS. The prediction capability of each classifier was measured by the area under receiver operating characteristic (ROC) curve (AUC) for discriminating the ROCK1 inhibitors from non-inhibitors.

Finally, each molecule in the ChemBridge database, which is extensively used in VS, was consequentially docked into eight crystal structures of ROCK1 and mapped onto eight pharmacophore models, and scored by the best Bayesian classifier. The drug-likeness of the top 100 compounds ranked by the Bayesian scores was evaluated by Lipinski’s “Rule-of-Five”^52^, the REOS (rapid elimination of swill) rules^53^, and the drug-likeness filter developed in our group^54^, and the non-druglike molecules were filtered out. Then, the remaining compounds were structurally clustered, and the compounds with the Tanimoto similarity coefficients computed from the *MACCS* structural keys higher than 0.85 were clustered into the same group^55^. Finally, 38 potential inhibitors of ROCK1 were purchased for *in vitro* ROCK1 inhibitory assay.

### Enzyme Activity Assay

Kinase inhibition was measured by using the Z′-LYTE™ Kinase Assay Kit with Ser/Thr 7 peptide substrate (Invitrogen, cat. PV3180). Compounds were tested on three individual times with eight point dilutions performed in duplicate to determine the average IC_50_ values. The assay conditions were optimized to 15 μl kinase reaction volume with 5 ng ROCK1 enzyme in 50 mM HEPES (pH = 7.5), 10 mM MgCl_2_, 0.01% Brij-35 and 1 mM EGTA. Initially, the reaction was incubated for 1 hour at room temperature in the presence of 1.5 μM peptide substrate and 12.5 μM ATP for ROCK1. Then, 5 μL development reagent A was added and the assay plate was incubated for another 1 hour. Finally, 5 mL stop reagent was added and the assay plate was shaken for 1 hour. The coumarin (ex. 400 nm, em. 445 nm) and fluorescein (ex. 400 nm, em. 520 nm) emission signals were measured on a fluorescence plate reader (Thermo Scientific Varioskan Flash).

### Substructure search

The substructure search function implemented in MOE^55^ was employed to find the analogues with the scaffold of *4-Phenyl-1H-pyrrolo [2,3-b] pyridine* from the ChemBridge database. Then, 41 analogues were identified and purchased for bioassays.

### Cell Culture

Human umbilical vein endothelial cells (HUVEC, ATCC, Manassas, USA) were cultured and maintained in the F-12 K medium with 2 mM L-glutamine, 1.5 g/L sodium bicarbonate, 100 μg/mL heparin, 30 μg/mL endothelial cell growth supplement and 10% fetal bovine serum (FBS) at 37 °C in a humidified atmosphere of 5% CO_2_. Cells were exposed to culture medium with 10% FBS for at least 1 day before experiments. Only 3–6 passages of cells were used in this study.

### Western Blotting Analysis

HUVEC was pretreated with different targeted compounds (TS-15 and TS-40) for 1 hour. Y27632 and 0.2% DMSO were used as the positive control and vehicle control, respectively. After drug treatment, cells were washed with pre-cold PBS and lysed on ice with RIPA lysis buffer for 30 min, followed by centrifugation at 12,500 g for 20 min at 4 °C. The supernatant was collected and protein concentration was determined using the BCA protein assay kit (Thermo Scientific Pierce). Aliquots of protein samples were boiled for 5 min at 95 °C and electrophoresed on 10% SDS-PAGE and then transferred to a polyvinylidene difluoride membrane (Bio-Rad, Hercules, CA, USA). Subsequently, the membrane was blocked with 5% (w/v) nonfat milk in TBS-T buffer for 1 hour at room temperature. The blots were incubated overnight at 4 °C with primary antibodies. After being washed with TBS-T for 20 min at room temperature, the membranes were further incubated with horseradish peroxidase-conjugated secondary antibodies for 2 hours at room temperature. Finally, protein bands were visualized using ECL Plus Western blotting detection reagents (GE Healthcare, Piscataway, NJ, USA). The membranes were then scanned on a Bio-Rad ChemiDoc imaging system and the intensity of the protein bands was analyzed using the Bio-Rad Image Lab software.

### Zebrafish Maintenance

Tg (fli1a:EGFP)y1; Tg (gata1a:dsRed)sd2 homozygous double transgenic zebrafish was used and maintained as described in our previous study^43^. The embryos were cultured at 28.0 °C in embryo medium (13.7 mM NaCl, 540 mM KCl, 25 mM Na_2_HPO_4_, 44 mM KH_2_PO_4_, 300 mM CaCl_2_, 100 mM MgSO_4_, 420 mM NaHCO_3_, pH = 7.4) according to the description in the Zebrafish Handbook. The animal experiments were carried out in “accordance” with the approved guidelines of the Animal Research Ethics Committee, University of Macau and approved by the Animal Research Ethics Committee, University of Macau.

### Atorvastatin-induced Cerebral Hemorrhage in Zebrafish

Tg (fli1a:EGFP)y1; Tg (gata1a:dsRed)sd2 homozygous double transgenic zebrafish embryos at 22 hours post-fertilization (hpf) were pretreated with the targeted compounds (TS 15 and TS 40) for 2 hours. During this period, the positive control group was treated with 10 mM Y27632 while the vehicle control group was given with 0.2% DMSO (solvent). Then, all the pretreated embryos washed with PBS for 3 times and challenged with 1 mM atorvastatin for 24 hours according to the reported protocol with small modifications^56^. After that, the viability and cerebral hemorrhage changes were observed using a fluorescence microscope (Olympus IX81 Motorized Inverted Microscope, Japan) equipped with a digital camera (DP controller, Soft Imaging System, Olympus, Germany). The results were analyzed with Axiovision 4.2 (Soft Imaging System, Olympus, Germany) and the public-domain Image J software (Rasband WS, Image J; US National Institutes of Health, Bethesda, MD). The cerebral hemorrhage rate of each group was calculated by the number of zebrafish with cerebral hemorrhage to the total number of each group.

## Results and Discussion

### Virtual Screening and ROCK1 Inhibitory Activity

**Figure 2:**
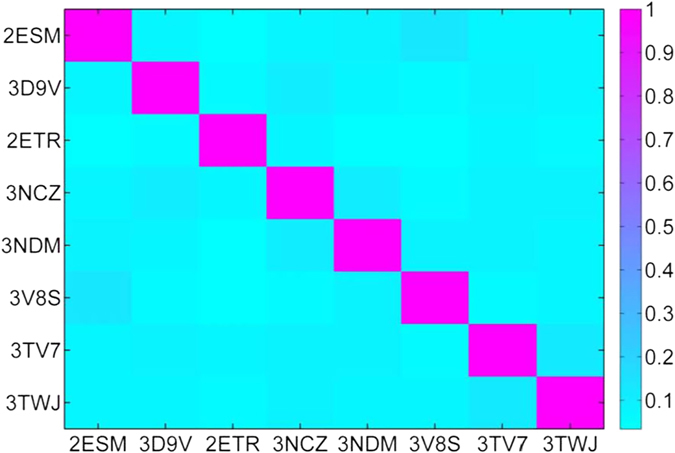
The similarity scores of *Assemblies* for the top 500 molecules ranked by the *Glide* docking scores based on eight crystal structures of ROCK1-ligand complexes.

As reported in our previous study^42^, eight out of nine crystal complexes of ROCK1 satisfied the requirements of “docking power” (RMSD ≤ 2.0 Å) and “distinguishing power” (*P*-value ≤ 10^−20^) by using either *Glide* SP or XP scoring. The top 500 hits ranked by the *Glide* docking scores based on eight different crystal structures of ROCK1 were compared based on the similarity scores of *Assemblies*. As shown in Fig. 2, the similarity score of the top 500 hits for any two different ROCK1-ligand crystal structures was lower than 0.15, suggesting that the top-scored hits given by molecular docking based on different crystal structures were quite different (Table S1 in Supporting Information). In other words, the potential inhibitors predicted by docking-based VS were dependent on the conformations of the target. Therefore, in this study, the NBC technique was employed to integrate the predictions from molecular docking and pharmacophore mapping based on eight crystal structures of ROCK1-ligand complexes. Compared with the classifiers only based on the docking scores or fit values derived from any single protein structure, the Bayesian classifiers based on the docking scores and fit values derived from eight ROCK1-ligand crystal structures performed much better. For the dataset with the known inhibitors and non-inhibitors of ROCK1, the best Bayesian classifier could achieve a sensitivity of 0.826, a specificity of 0.875, a global accuracy of 0.873 and an AUC value of 0.938 based on the docking scores and fit values derived from eight ROCK1-ligand crystal structures. Then, the best Bayesian classifier was utilized to screen the ChemBridge database for identifying potential inhibitors of ROCK1, and 38 potential inhibitors of ROCK1 were identified and purchased for ROCK1 inhibitory activity assay.

**Figure 3:**
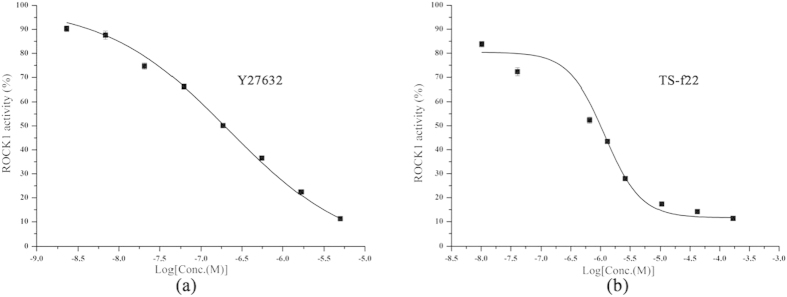
The concentration-dependent inhibition of ROCK1 activities for Y27632 and TS-f22.

**Figure 4:**
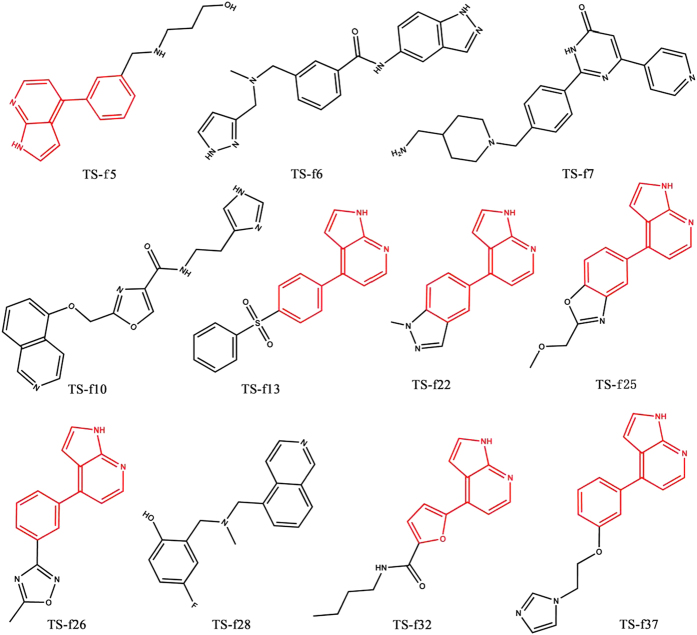
The chemical structures of eleven inhibitors of ROCK1 identified by integrated VS strategy and enzyme-based assay.

First, these compounds were evaluated in triplicate on three individual times at the concentration of 41.67 ug/ml. The results showed that 11 compounds had more than 50% inhibition activities of ROCK1. The hit rate was 28.95%, suggesting that the integrated VS strategy was quite reliable and accurate. Then, the IC_50_ values of these 11 compounds were determined on three individual times with 8-point dilutions. As shown in Table 1 and Fig. 3, seven compounds had the IC_50_ values lower than 10 μM and compound TS-f22 exhibited the most potent activity with IC_50_ = 480 nM. The chemical structures of the 11 promising ROCK1 inhibitors were shown in Fig. 4. In our previous study, 12 potent ROCK1 inhibitors (IC_50_ values between 7 and 28 μM) were discovered from 174 tested compounds given by molecular docking based on a single crystal structure of ROCK1^32^. That is to say, the hit rate reported in our previous study was only 6.90%, which was quite lower than that (28.95%) reported in this study.

Besides, it should be noted that some inhibitors identified by the integrated VS protocol could not be successfully predicted by molecular docking based on a single crystal structure of ROCK1. For example, TS-f5 and TS-f22 were the most potent inhibitors shown in Table 1. If 3TV7 and 2ETR were used as the templates for molecular docking, the ranks of the docking scores for TS-f5 were 5818 and 30558, and those for TS-f22 were 6815 and 3060, respectively (Table S3 in Supporting Information). If the top 1000 hits predicted by molecular docking based on 3TV7 or 2ETR were chosen for bioassays, TS-f5 and TS-ff22 even did not have any chance to be experimentally tested. Therefore, we believed that the integrated VS strategy based on multiple structures of ROCK1 had better capability to discover potent inhibitors than the traditional VS strategies based on a single structure of ROCK1.

### Structural Analysis of Potent ROCK1 Inhibitors

The structures of the identified ROCK1 inhibitors were compared with the known inhibitors of ROCK1 deposited in the BindingDB database^50^ by using the pairwise Tanimoto coefficients based on the ECFP_6 fingerprints calculated by using the *Find Similar Molecules by Fingerprints* protocol in DS3.1^51^. The statistical results illustrated that the identified ROCK1 inhibitors did not share high structural similarities with any known ROCK1 inhibitor. The highest structural similarity between the 11 identified inhibitors and known inhibitors of ROCK1 was below 0.4 (Table 1). Interestingly, seven compounds shared the same substructure, *4-Phenyl-1H-pyrrolo [2,3-b] pyridine* (highlighted in Fig. 4), which might be served as a novel chemical scaffold for ROCK1 inhibitors. Moreover, as shown in Table 1, all these inhibitors satisfied the drug-likeness rules defined by Qikprop^57^.

### Structure-Activity Relationships of the Analogues of TS-f22

In order to understand the structure-activity relationships (SARs) of the analogues of TS-f22 and discover more potent ROCK1 inhibitors with the scaffold of *4-Phenyl-1H-pyrrolo [2,3-b] pyridine*, the substructure search was employed to screen the ChemBridge database, and 41 analogues of TS-f22 were identified and purchased for bioassays. In the first round testing, ten out of the 41 compounds had more than 50% inhibition activities at the concentration of 41.67 ug/ml. Therefore, the IC_50_ values of these ten compounds were determined and listed in Table 2. Based on the experimental data shown in Table 2, preliminary SARs were discussed below.

Our initial array focused on the substitution on the pyrrolopyridine ring at R_1_ (Table 2). Astonishingly, most substitutions had negative contributions, including ring substitutions, amines and amides substitutions. The IC_50_ values of these weaker-binding compounds ranged from 4.47 to 67.2 μM. According to the binding structures predicted by molecular docking, the pyrrolopyridine ring bound into the ATP binding pocket of ROCK1, but the added substitution on the pyrrolopyridine ring might cause the conformational change of the pyrrolopyridine ring, thus impairing the interaction between the inhibitors and the ATP binding pocket.

**Figure 5:**
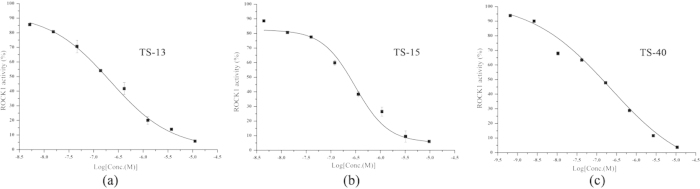
The concentration-dependent inhibition of ROCK1 activities for TS-13, TS-15 and TS-40.

Moreover, no substitution (H substitution) at R_1_ improved the potency obviously. All the H-substituted compounds had relatively high inhibitory activities (<10 μM) and three of them (TS-13, TS-15 and TS-40) showed excellent potencies with IC_50_ < 300 nM (Fig. 5). These results showed that the H substitution at R_1_ was favorable to achieve improved inhibitory activity against ROCK1.

Then, we examined the influence of the modifications at the *ortho*, *meta* or *para* position of the phenyl group (Table 2). The compounds (TS-13, TS-15, TS-24 and TS-40) sharing similar groups at R_1_ were compared. The comparison between TS-13 and TS-15 showed that the inhibitory activity was slightly affected by the modifications at the *para* position. Moreover, the substitution of hydrogen with a bulk goup at the *meta* group gave a 50-fold decrease in potency (compound TS-24).

**Figure 6:**
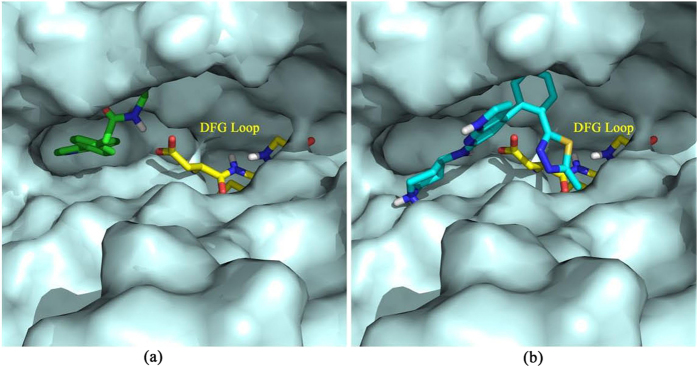
The binding pocket of ROCK1 represented by solvent-accessible surface for (a) TS-13 and (b) TS-20.

The SAR analyses showed that the pyrrolopyridine group without modification and the phenyl group with the substitution at the *para* position were favorable for ligand binding. In order to explain the SARs in detail, the binding structures of the 17 inhibitors with *4-Phenyl-1H-pyrrolo [2,3-b] pyridine* predicted by *Glide* (2ESM as the docking template) were analyzed. Compound TS-13 was one of the most promising inhibitors that could bind tightly into the active site of ROCK1. The pyrrolopyridine group of TS-13 was buried in the ATP binding site, and the ethyl phenylacetate group extended outside the ATP binding site and formed a hydrogen bond with Asp216 of the DFG loop (Fig. 6a), which could explain the strong inhibitory activity of TS-13. Compound TS-20 was one of the typical inhibitors that were modified at two positions: the *ortho* position of phenyl and the pyrrolopyridine group by adding an amino-piperidine group. As described in Fig. 6b, compared with TS-13, the added amino-piperidine group resulted in huge conformational change. The substituted pyrrolopyridine group was positioned in the space beyond the ATP active pocket, and therefore, there was no strong intermolecular interaction between TS-20 and the ATP binding site. Since there was no substitution at the R_4_ position, it could not form stable interaction with the DFG loop, thus leading to worse inhibitory activity.

**Figure 7:**
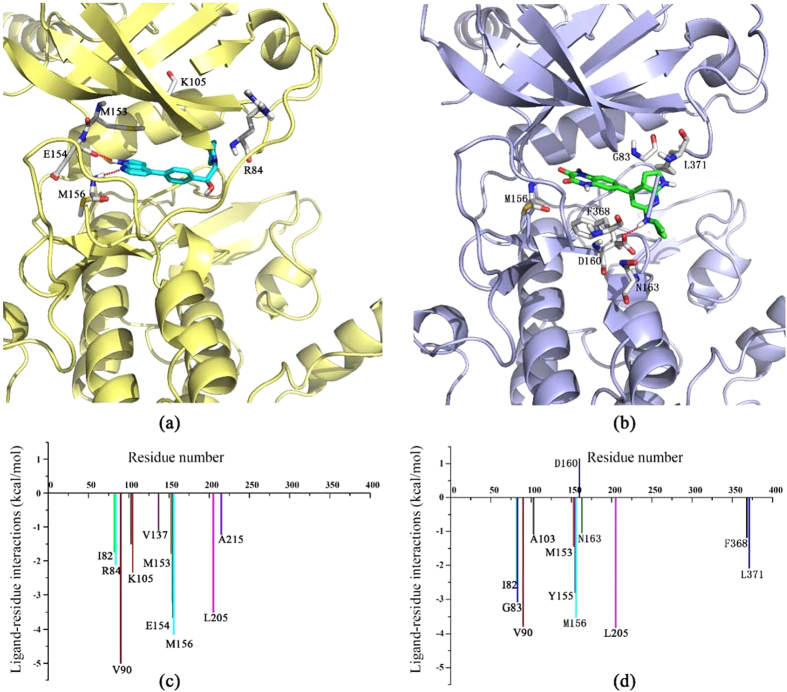
The averaged structures from the MD simulations for the (a) TS-15–ROCK1 complex and (b) TS-11–ROCK1 complex (carbon atoms of the key residues were colored in gray, and carbon atoms of the TS-15 and TS-11 were colored in cyan and green, respectively). The contributions of the key residues to the binding of (**c**) TS-15 and (**d**) TS-11.

In addition, the binding modes of the ROCK1/TS-11 and ROCK1/TS-15 complexes were compared and the interactions between the key residues and TS-11/TS-15 were analyzed by using the MM/GBSA free energy calculations and decompositions^49^,^51^,^55^,^57^,^58^,^59^,^60^,^61^ (the calculation details can be found in Supporting Information). TS-15 (IC_50_ = 250 nM) was one of the most potent inhibitors, which was composed of an original pyrrolopyridine group and a phenyl group modified at the *para* position. TS-11 (IC_50_ = 67.2 μM) was modified in both the pyrrolopyridine group and the phenyl group. As shown in Fig. 7a,b, the binding conformations of TS-11 and TS-15 predicted by the MD simulations were not well aligned in the active site of ROCK1, which might be also caused by the two modifications discussed above. The pyrrolopyridine group of TS-15 bound into the ATP binding site by forming two hydrogen bonds with Glu154 and Met156. However, the pyrrolopyridine group of TS-11 was outside the ATP binding site, and was stabilized by a hydrogen bond with Asp160. TS-15 had much better bioactivity than compound TS-11, and the difference of their predicted binding free energies was -8.11 kcal/mol. The key residues for the binding of TS-11 and TS-15 were compared (Fig. 7c,d). The residues Ile82, Val90, Met153, Met156 and Leu205 were favorable for the binding of both TS-11 and TS-15. However, the residues Glu154 and Arg84 played more important role in the binding of TS-15 than that of TS-11. Glu154 formed strong polar interaction (−3.20 kcal/mol) with the pyrrolopyridine group of TS-15 and Arg84 formed strong non-polar interaction (− 3.04 kcal/mol) with the cyclopentane group of TS-15.

### Effects of Inhibiting the Phosphorylation of Downstream Target of ROCK

Western blotting was then employed to investigate the effects of four ROCK1 inhibitors with IC_50_ < 1.0 μM (TS-f22, TS-13, TS-15 and TS-40) on the signaling pathway of ROCK in human umbilical vein endothelial cells (HUVEC). Cofilin has also been proven as an important downstream target in the ROCK pathways, and its phosphorylation level reflects the regulations of the novel inhibitors in the ROCK activity in cellular environment^22^,^43^,^62^. Moreover, cofilin is also one of the significant proteins that regulate actin remodeling^63^.

**Figure 8:**
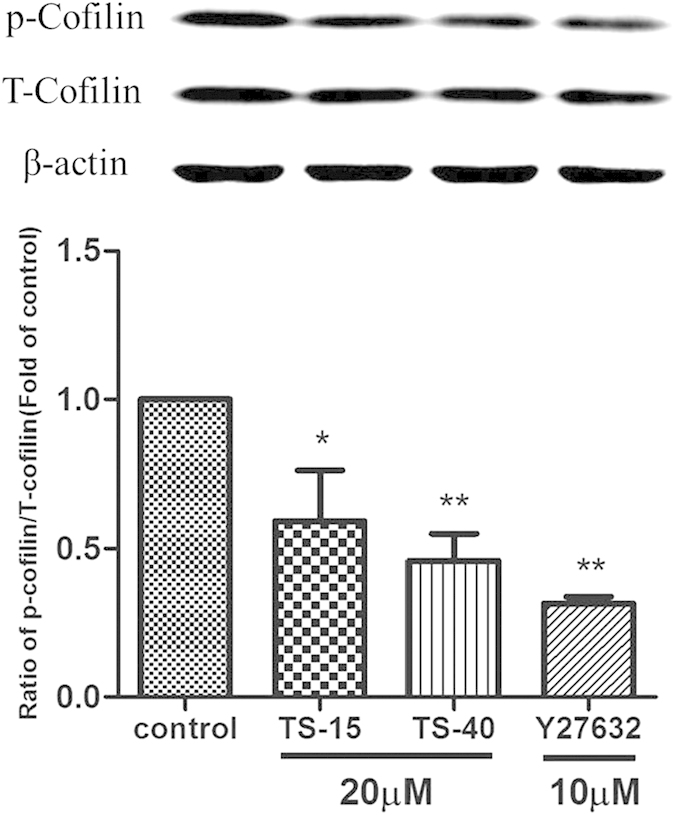
The down-regulations of TS-15 and TS-40 in the phosphorylations of cofilin in human umbilical vein endothelial cells. HUVEC was treated with Y27632, TS-15 or TS-40 for 2 hours. Then, the ratio of p-conflin to T-conflin of each group was normalized by the value the control. Data represented the mean ± RSD (*n* = 4). Statistical comparison of the data was performed by one-way ANOVA followed by Dunnett’s test. *P < 0.05 and **P < 0.01 compared to the control group.

Y27632, a representative ATP-competitive inhibitor of ROCKs, was used as the positive control. The results showed a strong cross-reactive band for p-cofilin in the control lane, indicating large amounts of p-cofilin (Fig. 8). With the treatment of Y27632, the phosphorylation level of cofilin was reduced significantly when compared with the control group. Similar to Y27632, HUVECs pre-treated with TS-15, TS-40 or Y27632 showed a faint band for p-cofilin, suggesting great inhibition of Rho kinase in the cells. Obviously, TS-15 and TS-40 had significant inhibitory effects on the phosphorylation of cofilin, and could down-regulate the ROCK pathways in HUVEC.

### Effects on Reducing Atorvastatin-induced Cerebral Hemorrhage in Zebrafish

**Figure 9:**
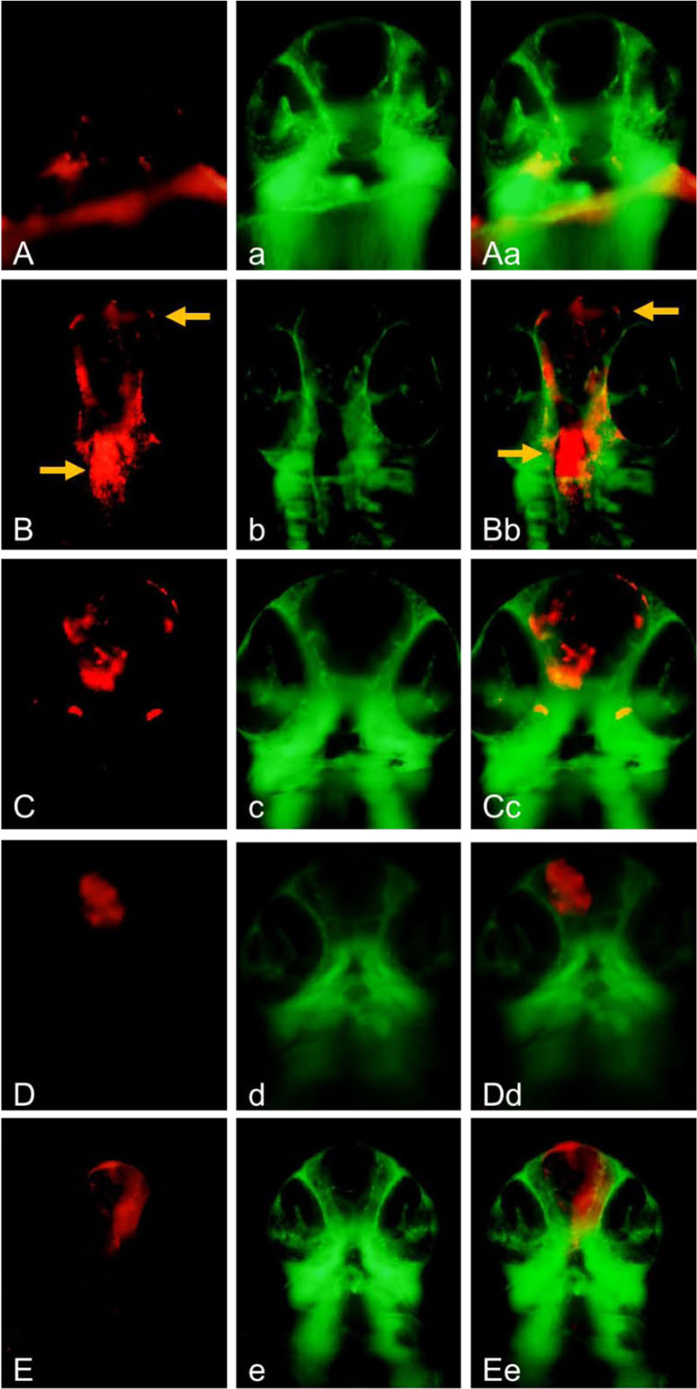
The protective effects of TS-15 and TS-40 against atorvastatin-induced cerebral hemorrhage in zebrafish. (**A,a** and **Aa**) the embryos treated with 0.2% DMSO served as the normal control group; (**B,b** and **Bb**; **C,c** and **Cc; D,d** and **Dd; E,e** and **Ee**) the embryos were pretreated with either 0.2% DMSO, Y27632 (10 mM), TS-15 (20 mM) or TS-40 (20 mM) for 2.0 hours and replaced with 1 mM atorvastatin for 24 hours. Homozygous double transgenic zebrafish, the red fluorescence was the Tg (gata1: dsRed) sd2 (**A**–**E**), the green fluorescence was Tg (fli1a: EGFP) y1 (**a**–**e**), and the third column was the overlapping photos of the first two columns (**Aa**, **Bb**, **Cc**, **Dd** and **Ee**). The arrows indicated the erythrocyte accumulation in cerebral hemorrhage region in zebrafish head.

Atorvastatin, a small molecule drug, has been reported to induce cerebral hemorrhage in zebrafish through the disruption of cell-cell junctions^43^,^62^. The inhibitory effects of TS-15 and TS-40 against cerebral bleeding were examined in this *in vivo* cerebral hemorrhage system. The healthy control group displayed normal phenotype without hemorrhage occurrence (Fig. 9A) while the atorvastatin-induced disease model group displayed significant cerebral hemorrhage (Fig. 9B). As for the positive control group and the other experimental groups, the hemorrhage changes were obviously abated by the pretreatment of Y27632 (Fig. 9C), TS-15 (Fig. 9D) or TS-40 (Fig. 9E), when compared with the disease model group.

Semi-quantitative analysis of the cerebral hemorrhage rate was presented in Table 3. As the positive control, the inhibitory rate of Y27632 against cerebral hemorrhage at the dose of 10 μM was 74.4%. The inhibitory rates of TS-15 and TS-40 against cerebral hemorrhage at the dose of 60 μM were < 60% and 66.2%, respectively, and those at the dose of 20 μM were 73.5% and 78.8%, respectively. Even at the dose of 6 μM, the inhibitory rate of TS-15 was 79.3%. However, TS-15 was toxic to zebrafish at the dose of 60 μM but relatively safe at the dose of 20 μM. These results demonstrated that, similar to Y27632, TS-15 or TS-40 functioned as a ROCK inhibitor and substantially suppressed atorvastatin-induced cerebral hemorrhage. However, as displayed in Table 3, ROCK1 inhibitors could alleviate but could not completely block the atorvastatin-induced bleeding, suggesting ROCK might not the only target for atorvastatin-induced cerebral hemorrhage. Besides, compared with Y27632, TS-15 or TS-40 had weaker inhibitory effect against cerebral hemorrhage, suggesting that the optimization of our inhibitors in the near future was still necessary.

## Conclusion

In this study, among the 79 compounds identified by the integrated VS strategy and substructure search, 21 showed obvious ROCK1 inhibitory activities. In total, 14 hits have the IC_50_ values below 10 μM, and four below 500 nM, highlighting the high prediction accuracy of the integrated VS strategy. Besides, the SARs analysis showed that the pyrrolopyridine group without modification and the phenyl group with the substitution at the *para* position were favorable to achieve better activities against ROCK1. In addition, two inhibitors (TS-15 and TS-40) were proven effective in inhibiting the phosphorylation of the downstream target in the ROCK signaling pathway *in vitro* and protecting atorvastatin-induced cerebral hemorrhage *in vivo*. The integrated VS strategy employed in this study can be used as a powerful tool for identifying promising active compounds for targets of interest.

## Additional Information

**How to cite this article**: Shen, M. *et al.* Discovery of Novel ROCK1 Inhibitors *via* Integrated Virtual Screening Strategy and Bioassays. *Sci. Rep.* **5**, 16749; doi: 10.1038/srep16749 (2015).

## Supplementary Material

Supplementary Information

## Figures and Tables

**Table 1:** Experimentally determined half-maximal inhibitory concentrations (IC_50_) for Y27632 and the 11 inhibitors identified by the integrated VS strategy and the properties predicted by QikProp.

No.	IC_50_ (μM)	MW^a^	log*P*^b^	log*S*^c^	*P*_Caco_^d^	Similarity^e^
Y27632	0.17 ± 0.01	247.34	1.46	−2.28	218.77	0.39
TS-f5	1.55 ± 0.57	281.36	2.34	−2.88	160.88	0.34
TS-f6	12.4 ± 0.85	360.22	2.17	−3.65	59.20	0.36
TS-f7	28.3 ± 0.99	375.48	1.73	−3.17	12.75	0.20
TS-f10	31.35 ± 1.63	363.38	2.20	−4.33	352.91	0.21
TS-f13	3.2 ± 1.060	334.40	3.27	−4.69	602.73	0.41
TS-f22	0.48 ± 0.06	248.29	3.38	−4.36	1548.30	0.36
TS-f25	4.42 ± 1.14	279.30	2.66	−3.64	1633.03	0.35
TS-f26	3.48 ± 1.65	276.30	2.78	−4.49	687.51	0.36
TS-f28	23.05 ± 0.07	296.13	3.11	−2.97	560.11	0.31
TS-f32	2.21 ± 0.21	283.33	3.17	−4.78	804.27	0.41
TS-f37	1.82 ± 0.76	304.35	3.97	−5.03	1436.03	0.34

^a^Molecular weight.

^b^predicted octanol/water partition coefficient.

^c^predicted aqueous solubility, *S* in mol/L.

^d^predicted Caco-2 cell permeability in nm/s (<25, poor;>500, great).

^e^pairwise Tanimoto similarity indices based on the ECFP_6 fingerprints for each inhibitor with the known ROCK1 inhibitors.

**Table 2:**
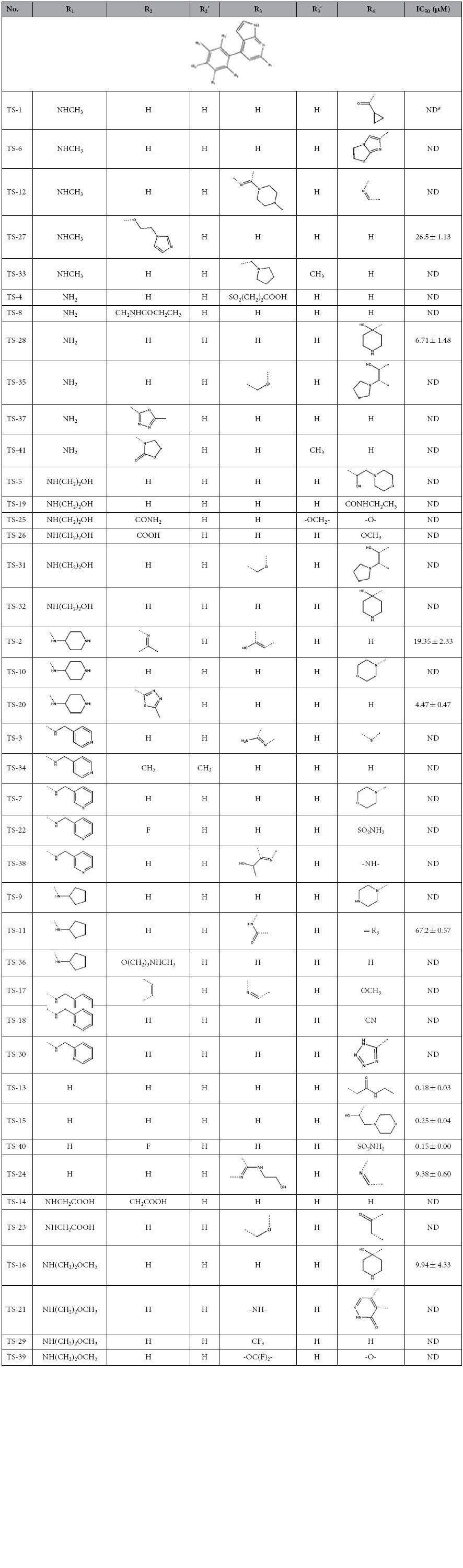
Structures and experimentally determined half-maximal inhibitory concentrations (IC_50_) for the 41 analogues of TS-f22.

^a^ND represents not determined.

**Table 3:** The protective effects of compound TS-15 and TS-40 against chemically-induced cerebral hemorrhage in zebrafish.

Compounds	Cerebral hemorrhage rate (%)^a^
Control	100
TS-15	
60 μM	<60^b^,*
2 0μM	73.5 ± 13.0*
6 μM	79.3 ± 5.9
TS-40	
60 μM	66.2 ± 7.2*
20 μM	78.8 ± 7.1*
6 μM	90.6 ± 7.4
Y27632	
10 μM	74.4 ± 8.7*

^a^The cerebral hemorrhage rate of each group was calculated by the number of zebrafish with cerebral hemorrhage symptoms to the total number of each group (10 embryos per group);

^b^Obvious mortality and acute toxicity were observed. Data represented the mean ± RSD (*n* = 4). Statistical comparison of the data was performed by one-way ANOVA followed by Dunnett’s test. *P < 0.05 compared to the control group.
