# Supplementary material for: Discovery of Novel ROCK1 Inhibitors via Integrated Virtual Screening Strategy and Bioassays
Source: Sci Rep. 2015 Nov 16;5:16749. doi: 10.1038/srep16749 (PMC4645114; doi:10.1038/srep16749)
Supplement: Supplementary Information [file srep16749-s1.pdf]

Discovery of Novel ROCK1 Inhibitors *via* Integrated Virtual Screening Strategy and Bioassays

Mingyun Shen[a,b#], Sheng Tian[b#], Peichen Pan[a], Huiyong Sun[a], Dan Li[a], Youyong Li[b], Hefeng Zhou[c], Chuwen Li[c], Simon Ming-Yuen Lee[c*], Tingjun Hou[a*]

[a]College of Pharmaceutical Sciences, Zhejiang University, Hangzhou, Zhejiang 310058, China

[b]Institute of Functional Nano & Soft Materials (FUNSOM), Soochow University, Suzhou, Jiangsu 215123, China

[c]State Key Laboratory for Quality Research in Chinese Medicine and Institute of Chinese Medical Sciences, University of Macau, MO 999078, China

Supporting Information

Part1. Molecular Dynamics (MD) Simulations

The dynamic interaction patterns between ROCK1 and two inhibitors (compounds TS-11 and TS-15) with novel scaffolds were investigated by the MD simulations. The general AMBER force field (*gaff*)[1] and the ff99SBildn force field[2] were used for the inhibitors and ROCK1, respectively. Each inhibitor was optimized by Gaussian 03at the HF/6-31G* level,[3] and then the atomic partial charges were obtained by fitting the electrostatic potentials using the RESP fitting technique in Amber11.[4]The system was neutralized with the counter ions of Na+. The whole system was immersed in a rectangular box of TIP3P water molecules,[5] and the water box was extended 12 Å from any solute atom. The particle mesh Ewald (PME) method was employed for the long-range electrostatics.[6]

Each system was relaxed by two-stage minimization protocol: first, the protein was fixed, the water molecules and ligand were minimized by 500 cycles of steepest descent and 500 cycles of conjugate gradient minimization; second, the whole system

was minimized by 5000 cycles (1000 cycles of steepest descent and 4000 cycles of conjugate gradient minimization). Then, 10 ns MD simulation was performed under a target temperature of 300 K and a target pressure of 1 atm. The SHAKE procedure was employed to constrain all bonds involving hydrogen atoms, and the time step was set to 2 fs.[7] Coordinate trajectories were saved every 10 ps. The MM optimization and MD simulations were accomplished by using the *sander* program in AMBER11.[8]

Part 2. MM/GBSA Binding Free Energy Calculations and Decomposition

The binding free energy for each inhibitor was calculated by the MM/GBSA approach (see Eq. 1):[9-20]

$$\begin{aligned}\Delta G_{bind} &= G_{complex} - G_{protein} - G_{ligand} \\ &= \Delta H + \Delta G_{solvation} - T\Delta S \\ &= \Delta E_{MM} + \Delta G_{GB} + \Delta G_{SA} - T\Delta S\end{aligned} \quad (1)$$

where ΔE_{MM} represents the gas-phase interaction energy between protein and ligand, which contains the electrostatic (ΔE_{ele}) and van der Waals (ΔE_{vdw}) terms; ΔG_{GB} and ΔG_{SA} is the polar and non-polar terms of desolvation free energy, respectively. Because of the expensive computational cost and low prediction accuracy,[21] the change of conformational entropy ($-T\Delta S$) upon ligand binding was ignored in our work. The electrostatic solvation energy (ΔG_{GB}) was calculated by using the GB model with the parameters developed by Onufriev*et al.* (*igb*=2).[22]The exterior dielectric constant was set to 80, and the solute dielectric constant value was set to 1. The non-polar contribution of desolvation (ΔG_{SA}) was estimated by solvent accessible surface area using the LCPO method.[23] All energy components were calculated using 100 snapshots extracted from 2.0 to 10 ns.

MM/GBSA free energy decomposition in the *mm_pbsa* program in AMBER11 was employed to analyze the interactions between each residue in ROCK1 and each inhibitor. The residue-inhibitor interaction consists of four parts, van der Waals contribution (ΔG_{vdw}), electrostatic contribution (ΔG_{ele}), the polar part of desolvation (ΔG_{GB}), and the non-polar part of desolvation (ΔG_{SA}). The exterior dielectric constant

was set to 80, and the solute dielectricconstant value was set to 1. The non-polar contribution of desolvation (ΔG_{SA}) was calculated by SASA using the ICOSA technique.[24]

Table S1. The similarity scores of *Assemblies* for the top 500 hits ranked by the docking scores for eight ROCK1-ligand complexes.

PDB ID	2ESM	3D9V	2ETR	3NCZ	3NDM	3V8S	3TV7	3TWJ
2ESM	1	0.067	0.039	0.077	0.085	0.128	0.075	0.069
3D9V	0.067	1	0.052	0.108	0.073	0.052	0.083	0.071
2ETR	0.039	0.052	1	0.067	0.049	0.034	0.078	0.058
3NCZ	0.077	0.108	0.067	1	0.104	0.051	0.091	0.082
3NDM	0.085	0.073	0.049	0.104	1	0.090	0.090	0.071
3V8S	0.128	0.052	0.034	0.051	0.090	1	0.060	0.077
3TV7	0.075	0.083	0.078	0.091	0.090	0.060	1	0.124
3TWJ	0.069	0.071	0.058	0.082	0.071	0.077	0.124	1

Table S2. The number, source of database, IDs and docking scores based on 2ESM as the docking template for the 17 experimentally identified inhibitors of ROCK1 with 4-Phenyl-1H-pyrrolo [2,3-b] pyridine.

No.	*Glide* docking score	Source of database	IDs
TS-f5[a]	-10.39	ChemBridge	17079087
TS-f13	-9.09	ChemBridge	33954726
TS-f22	-8.91	ChemBridge	53514674
TS-f25	-8.92	ChemBridge	60551779
TS-f26	-8.67	ChemBridge	61438106
TS-f32	-8.57	ChemBridge	78167004
TS-f37	-9.08	ChemBridge	95060900
TS-2	-4.99	ChemBridge	14708351
TS-11	-5.62	ChemBridge	32139845
TS-13	-9.27	ChemBridge	39706202
TS-15	-8.87	ChemBridge	43843524
TS-16	-5.30	ChemBridge	47993990
TS-20	-6.48	ChemBridge	48942215
TS-24	-5.89	ChemBridge	53372679
TS-27	-4.57	ChemBridge	59417029
TS-28	-6.43	ChemBridge	62716075
TS-40	-9.10	ChemBridge	95634477

[a]According to the purity statements, the purity of all substances purchased from ChemBridge is higher than 95%;

Table S3. The docking scores of TS-f5 and TS-f22 predicted by eight ROCK1-ligand complexes and the ranks of their docking scores predicted by using 3TV7 and 2ETR as the docking templates.

	2ESM	3D9V	2ETR	3NCZ	3NDM	3V8S	3TV7	3TWJ	$Rank_{3TV7}$	$Rank_{2ETR}$
TS-f5	-8.73	-8.78	-7.17	-9.64	-9.05	-8.35	-8.93	-8.76	5818	30558
TS-f22	-8.19	-7.49	-8.20	-8.73	-8.47	-9.55	-8.82	-8.60	6815	3060

References:

1. Wang, J. M.; Wolf, R. M.; Caldwell, J. W.; Kollman, P. A.; Case, D. A. Development and testing of a general amber force field. *J Comput Chem* **2004,** 25, 1157-1174.
2. Hornak, V.; Abel, R.; Okur, A.; Strockbine, B.; Roitberg, A.; Simmerling, C. Comparison of multiple Amber force fields and development of improved protein backbone parameters. *Proteins-Structure Function and Bioinformatics* **2006,** 65, 712-725.
3. Frisch, M. J.; Trucks, G. W.; Schlegel, H. B.; Scuseria, G. E.; Robb, M. A.; Cheeseman, J. R.; Montgomery, J. A. J.; Vreven, T.; Kudin, K. N.; Burant, J. C.; Millam, J. M.; Lyengar, S. S.; Tomasi, J.; Barone, V.; Mennucci, B.; Cossi, M.; Scalmani, G.; Rega, N.; Petersson, G. A.; Nakatsuji, H.; Hada, M.; Ehara, M.; Toyota, K.; Fukuda, R.; Hasegawa, J.; Ishida, M.; Nakajima, T.; Honda, Y.; Kitao, O.; Nakai, H.; Klene, M.; Li, X.; Knox, J. E.; Hratchian, H. P.; Cross, J. B.; Bakken, B.; Adamo, C.; Jaramillo, J.; Gomperts, R.; Stratmann, R. E.; Yazyev, O.; Austin, A. J.; Cammi, R.; Pomelli, C.; Ochterski, J. W.; Ayala, P. Y.; Morokuma, K.; Voth, G. A.; Salvador, P.; Dannenberg, J. J.; Zakrzewski, V. G.; Dapprich, S.; Daniels, A. D.; Strain, M. C.; Farkas, O.; Malick, D. K.; Rabuck, A. D.; Raghavachari, K.; Foresman, J. B.; Ortiz, J. V.; Cui, Q.; Baboul, A. G.; Clifford, S.; Cioslowski, J.; Stefanov, B. B.; Liashenko, A.; Liashenko, A.; Piskorz, P.; Komaromi, I.; Martin, R. L.; FOx, D. J.; Keith, T.; Al-Laham, M. A.; Peng, C. Y.; Nanayakkara, A.; Challacombe, M.; Gill, P. M. W.; Johnson, B.; Chen, W.; Wong, M. W.; Gonzalez, C.; Pople, J. A. Gaussian 03, Gaussian Inc.; Wallingford CT. **2004**.
4. Bayly, C. I.; Cieplak, P.; Cornell, W. D.; Kollman, P. A. A Well-Behaved Electrostatic Potential Based Method Using Charge Restraints for Deriving Atomic Charges - the Resp Model. *Journal of Physical Chemistry* **1993,** 97, 10269-10280.
5. Jorgensen, W. L.; Chandrasekhar, J.; Madura, J. D.; Impey, R. W.; Klein, M. L. Comparison of Simple Potential Functions for Simulating Liquid Water. *J Chem Phys* **1983,** 79, 926-935.
6. Darden, T.; York, D.; Pedersen, L. Particle Mesh Ewald - an N.Log(N) Method for Ewald Sums in Large Systems. *J Chem Phys* **1993,** 98, 10089-10092.
7. Ryckaert, J. P.; Ciccotti, G.; Berendsen, H. J. C. Numerical integration of the cartesian equations of motion of a system with constraints: molecular dynamics of n-alkanes. *Journal of Computational Physics* **1977,** 23, 327-341.
8. Case, D. A.; Cheatham, T. E.; Darden, T.; Gohlke, H.; Luo, R.; Merz, K. M.; Onufriev, A.; Simmerling, C.; Wang, B.; Woods, R. J. The Amber biomolecular simulation programs. *J Comput Chem* **2005,** 26, 1668-1688.
9. Wang, J. M.; Hou, T. J.; Xu, X. J. Recent advances in free energy calculations with a

combination of molecular mechanics and continuum models. *Curr. Comput.-Aided Drug Des.* **2006,** 2, 287-306.
10. Hou, T.; Yu, R. Molecular dynamics and free energy studies on the wild-type and double mutant HIV-1 protease complexed with amprenavir and two amprenavir-related inhibitors: mechanism for binding and drug resistance. *J. Med. Chem.* **2007,** 50, 1177-1188.
11. Liu, H.; Yao, X.; Wang, C.; Han, J. In silico identification of the potential drug resistance sites over 2009 influenza A (H1N1) virus neuraminidase. *Mol. Pharmaceut.* **2010,** 7, 894-904.
12. Zhang, J.; Hou, T.; Wang, W.; Liu, J. S. Detecting and understanding combinatorial mutation patterns responsible for HIV drug resistance. *Proc. Natl. Acad. Sci. USA* **2010,** 107, 1321-1326.
13. Hou, T. J.; Wang, J.; Li, Y. Y.; Wang, W. Assessing the performance of the molecular mechanics/Poisson Boltzmann surface area and molecular mechanics/generalized Born surface area methods. II. The accuracy of ranking poses generated from docking. *J. Comput. Chem.* **2011,** 32, 866-877.
14. Hou, T. J.; Wang, J. M.; Li, Y. Y.; Wang, W. Assessing the performance of the MM/PBSA and MM/GBSA methods. 1. The accuracy of binding free energy calculations based on molecular dynamics simulations. *J. Chem. Inf. Model.* **2011,** 51, 69-82.
15. Xue, W. W.; Pan, D. B.; Yang, Y.; Liu, H. X.; Yao, X. J. Molecular modeling study on the resistance mechanism of HCV NS3/4A serine protease mutants R155K, A156V and D168A to TMC435. *Antiviral Res.* **2012,** 93, 126-137.
16. Homeyer, N.; Gohlke, H. Free Energy Calculations by the Molecular Mechanics Poisson-Boltzmann Surface Area Method. *Mol. Inf.* **2012,** 31, 114-122.
17. Kollman, P. A.; Massova, I.; Reyes, C.; Kuhn, B.; Huo, S.; Chong, L.; Lee, M.; Lee, T.; Duan, Y.; Wang, W. Calculating structures and free energies of complex molecules: combining molecular mechanics and continuum models. *Accounts Chem. Res.* **2000,** 33, 889-897.
18. Huo, S.; Wang, J.; Cieplak, P.; Kollman, P. A.; Kuntz, I. D. Molecular dynamics and free energy analyses of cathepsin D-inhibitor interactions: insight into structure-based ligand design. *J. Med. Chem.* **2002,** 45, 1412-1419.
19. Hou, T. J.; Zhu, L. L.; Chen, L. R.; Xu, X. J. Mapping the binding site of a large set of quinazoline type EGF-R inhibitors using molecular field analyses and molecular docking studies. *J Chem Inf Comp Sci* **2003,** 43, 273-287.
20. Kuhn, B.; Gerber, P.; Schulz-Gasch, T.; Stahl, M. Validation and use of the MM-PBSA approach for drug discovery. *J. Med. Chem.* **2005,** 48, 4040-4048.
21. Hou, T.; Wang, J.; Li, Y.; Wang, W. Assessing the Performance of the MM/PBSA and MM/GBSA Methods. 1. The Accuracy of Binding Free Energy Calculations Based on Molecular Dynamics Simulations. *J. Chem. Inf. Model.* **2011,** 51, 69-82.
22. Onufriev, A.; Bashford, D.; Case, D. A. Exploring protein native states and large-scale conformational changes with a modified generalized born model. *Proteins-Structure Function and Bioinformatics* **2004,** 55, 383-394.
23. Weiser, J.; Shenkin, P. S.; Still, W. C. Approximate atomic surfaces from linear combinations of pairwise overlaps (LCPO). *J. Comput. Chem.* **1999,** 20, 217-230.
24. Gohlke, H.; Kiel, C.; Case, D. A. Insights into protein-protein binding by binding free

energy calculation and free energy decomposition for the Ras-Raf and Ras-RaIGDS complexes. *J. Mol. Biol.* **2003,** 330, 891-913.
